# *Varroa destructor* feeds primarily on honey bee fat body tissue and not hemolymph

**DOI:** 10.1073/pnas.1818371116

**Published:** 2019-01-15

**Authors:** Samuel D. Ramsey, Ronald Ochoa, Gary Bauchan, Connor Gulbronson, Joseph D. Mowery, Allen Cohen, David Lim, Judith Joklik, Joseph M. Cicero, James D. Ellis, David Hawthorne, Dennis vanEngelsdorp

**Affiliations:** ^a^Department of Entomology, University of Maryland, College Park, MD 20742;; ^b^Agricultural Research Service, Systematic Entomology Laboratory, United States Department of Agriculture, Beltsville, MD 20705;; ^c^Agricultural Research Service, Soybean Genomics & Improvement Laboratory, Electron and Confocal Microscopy Unit, United States Department of Agriculture, Beltsville, MD 20705;; ^d^Agricultural Research Service, Floral and Nursery Plant Research Unit, Electron and Confocal Microscopy Unit, United States Department of Agriculture, Beltsville, MD 20705;; ^e^Department of Entomology and Plant Pathology, North Carolina State University, Raleigh, NC 27695;; ^f^Entomology and Nematology Department, University of Florida, Gainesville, FL 32611

**Keywords:** *Varroa*, apiculture, insect physiology, honey bee, fat body

## Abstract

The parasitic mite *Varroa destructor* is the greatest single driver of the global honey bee health decline. Better understanding of the association of this parasite and its host is critical to developing sustainable management practices. Our work shows that this parasite is not consuming hemolymph, as has been the accepted view, but damages host bees by consuming fat body, a tissue roughly analogous to the mammalian liver. Both hemolymph and fat body in honey bees were marked with fluorescent biostains. The fluorescence profile in the guts of mites allowed to feed on these bees was very different from that of the hemolymph of the host bee but consistently matched the fluorescence profile unique to the fat body. Via transmission electron microscopy, we observed externally digested fat body tissue in the wounds of parasitized bees. Mites in their reproductive phase were then fed a diet composed of one or both tissues. Mites fed hemolymph showed fitness metrics no different from the starved control. Mites fed fat body survived longer and produced more eggs than those fed hemolymph, suggesting that fat body is integral to their diet when feeding on brood as well. Collectively, these findings strongly suggest that *Varroa* are exploiting the fat body as their primary source of sustenance: a tissue integral to proper immune function, pesticide detoxification, overwinter survival, and several other essential processes in healthy bees. These findings underscore a need to revisit our understanding of this parasite and its impacts, both direct and indirect, on honey bee health.

## Significance

*Varroa destructor* causes considerable damage to honey bees and subsequently the field of apiculture through just one process: feeding. For five decades, we have believed that these mites consume hemolymph like a tick consumes blood, and that *Varroa* cause harm primarily by vectoring viruses. Our work shows that they cause damage more directly. *Varroa* externally digest and consume fat body tissue rather than blood. These findings explain the failure of some previous attempts at developing effectively targeted treatment strategies for *Varroa* control. Furthermore, it provides some explanation for the diverse array of debilitating pathologies associated with *Varroa* that were unexplained by hemolymph removal alone. Our work provides a path forward for the development of novel treatment strategies for *Varroa*.

The parasitic mite *Varroa destructor* (*Varroa*) is the most significant single driver of managed European honey bee (*Apis mellifera*) colony losses worldwide (1). Several factors contribute to the dramatic effect of *Varroa* on honey bee populations, including the direct impact of their feeding on immature bees, their status as a confirmed vector of 5 debilitating viruses and potentially 13 others, their near ubiquitous presence in *A. mellifera* colonies, and the naïve nature of the host and parasite to one another due to their lack of historical sympatry (1–3). These factors have been studied extensively over the last half century but the conclusion that *Varroa* feed exclusively on the hemolymph of honey bees (hemolymphagy) has received little discernible scrutiny. Notably, multiple studies have been undertaken to explain the diverse array of honey bee pathologies associated with *Varroa* feeding that cannot be attributed to the removal of a small volume of hemolymph (4–11). These pathologies range from diminished immune function and reduced pesticide tolerance to impaired pupal development and shortened lifespan, underscoring an imperative to understand exactly how parasitic feeding impacts an insect critical to global food security (1, 5, 7, 8, 10, 12).

While vertebrate blood-feeding (hemophagy) is well documented in arthropods, the substantially lower nutrient content of insect hemolymph calls into question the ability of an organism to sustain itself exclusively on this resource (13–15). Vertebrate blood has a cell content of ∼40% by volume contributing to a relatively high nutrient content. Insect hemolymph generally consists of less than 2% cell content and has a generally dilute nutrient profile (16, 17). In line with these facts, the concept of hemolymphagy as the sole or main nutrient acquisition strategy in insects has been addressed and largely refuted (13–15, 18–20).

Our current understanding of *Varroa* feeding behavior is based on work conducted in the 1970s in which investigators used radioisotopes, [^90^Sr] or [^3^H], to conclude that the mites feed on their host’s hemolymph (21–23). [^3^H] lacks consistency as a marker for targeting specific tissues and has since fallen out of favor (22, 23). [^90^Sr] was used as a marker for hemolymph because of its tendency to replace calcium in tissue but the abundance of calcium in both hemolymph and fat body makes it impossible to identify a host meal in the presence of both tissues via this method. The issues with using either isotope for this purpose would be further exacerbated if *Varroa* employed extraoral digestion like most mesostigmatids, because liquid and semisolid tissues would dissolve together in the host before consumption. Attempts to develop systemic chemotherapeutics during the same period (miticides fed to the bees and consumed by the mites when they feed) consistently ended in failure, and as such, none are currently commercially available (1, 24). This is expected if the target host tissue has not been accurately identified. However, despite the failings of the experimental evidence and the existence of circumstantial evidence suggesting that this parasite feeds on a different host tissue, *Varroa* hemolymphagy has remained the accepted view. One reason for this persistence is likely the standard protocol of removing hemolymph samples from late-stage larvae and early-stage pupae, a time when large deposits of fat body are present in the hemolymph tissue. If the fat body is not separated carefully from the hemolymph, the molecular/nutritional contents of what is being called hemolymph strongly reflect the molecular/nutritional content of a commingled tissue that is largely fat body (25–27).

*Varroa* are closely allied phylogenetically with predatory and parasitic mites, many of which feed by extraoral digestion (28). This implies that they may consume semisolid tissue near where they attach to their host. Furthermore, the digestive system and mouthparts of this mite are structured in ways we would expect from an organism that feeds on semisolid tissue via extraoral digestion rather than hemolymph. For example, *Varroa* have a simple, tube-like gut structuring with no enzymatic activity in the midgut, and a gnathosoma (mouthparts) with well-developed salivary stylets allowing salivary fluid to mix efficiently with internal host tissue (24, 29). While *Varroa* possess attributes associated with feeding on semisolid tissue, they lack essential adaptations associated with hemolymph feeding. The evolutionary transition to dilute fluid feeding is usually accompanied by a reduction in sclerotization (chemical change resulting in stiffening) of the idiosoma (the body of the mite save the mouthparts and palps), which allows for the parasite to stretch to accommodate a large volume of liquid food (30). *Varroa* are conspicuously lacking this adaptation, along with a characteristic restructuring of the digestive system (i.e., a filter chamber or analogous adaptation) to manage the added osmoregulatory burden of a low-nutrient, high water diet (19, 24, 31–33). Furthermore, the waste product excreted by this mite (i.e., guanine) is usually associated with organisms that have an abundance of protein in their diet and significant water limitation (34).

Considering the discrepancies between *Varroa* physiology and what we would expect from a hemolymphage, we have proposed an alternative hypothesis that *Varroa* feed on honey bee fat body tissue. Such a feeding behavior is more consistent with our current understanding of the morphology and physiology of *Varroa* and the diversity of pathologies associated with its feeding. Fat body is a nutrient rich, vital organ near the cuticle of both adult and immature honey bees (35). It is the primary site of protein and urate synthesis and contains relatively high levels of guanine (35, 36). The essential role of the fat body in hormone regulation, immune response, and especially pesticide detoxification makes an understanding of the relationship between this parasite and this tissue particularly relevant to ongoing discussions on the causes of honey bee health decline (35). Moreover, determination of the primary source of nutrition for *Varroa* would change our understanding of the etiology of this parasite and could potentially lead to the development of novel—and of active interest—more effective treatment strategies (e.g., systemic pesticide formulations, interfering RNA, and so forth). To that aim, we conducted a three-tiered study asking the following questions: (*i*) Do so called phoretic *Varroa* feed primarily or exclusively in proximity to the fat body? (*ii*) What host tissue(s) is (are) being ingested by *Varroa* when feeding? (*iii*) What host tissue(s) is (are) integral to survivorship and reproduction in foundress mites?

To answer the first question, we conducted an observational study wherein we mapped the location of *Varroa* parasitizing adult bees. We hypothesized that the mites would not randomly disperse on adult bees, but rather, would be found preferentially at sites on the bee that maximized their ability to access their target food source. Fat body is distributed throughout the hemocoel of larvae and early-stage pupae but in adult bees it is primarily localized to the inner dorsal and ventral surfaces of the metasoma (the designation for the abdominal region in Hymenoptera) (37). Previous work detailing the feeding preferences of *Varroa* throughout brood development has shown that the mites show no preference in where to feed until late in the development of the pupa; a period characterized by the relocation of the fat body to the floor and roof of the abdomen (37, 38). This behavior suggests an association between *Varroa* and fat body in brood and would suggest the same in adult bees if *Varroa* continue feeding only in this location on worker bees.

## Results and Discussion

**Fig. 1.**
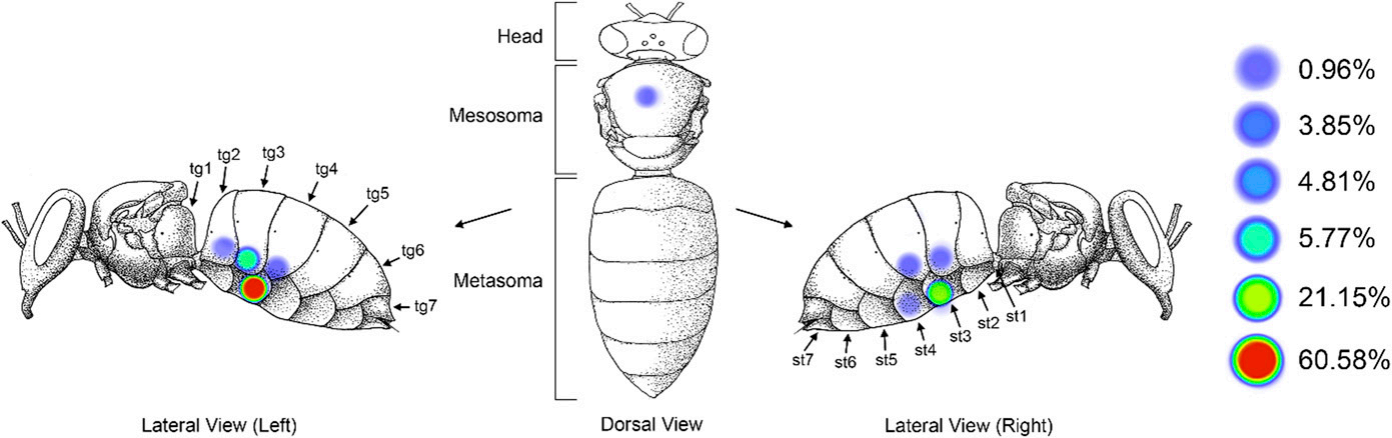
*V. destructor* shows consistent preference for the underside of the metasoma of adult host bees, an area predominated by fat body tissue just beneath the cuticle. (*Left*) Diagram showing frequency of *Varroa* found in each location on 104 parasitized worker bees in five trials (st, sternite; tg, tergite). *Varroa* were found on the underside of metasoma as opposed to locations on the mesosoma or head (generalized linear model, GLM: Χ_*2*_^*2*^ = 6.5, *P* < 0.001). Mites strongly preferred the third segment of the metasoma to any of the other 23 locations (GLM: Χ_*2*_^*2*^ = 4.5, *P* < 0.001). Mites were also found preferentially on the *Left* side of the host (χ): χ^*2*^ = 24.02, *P* < 0.001.

**Fig. 2.**
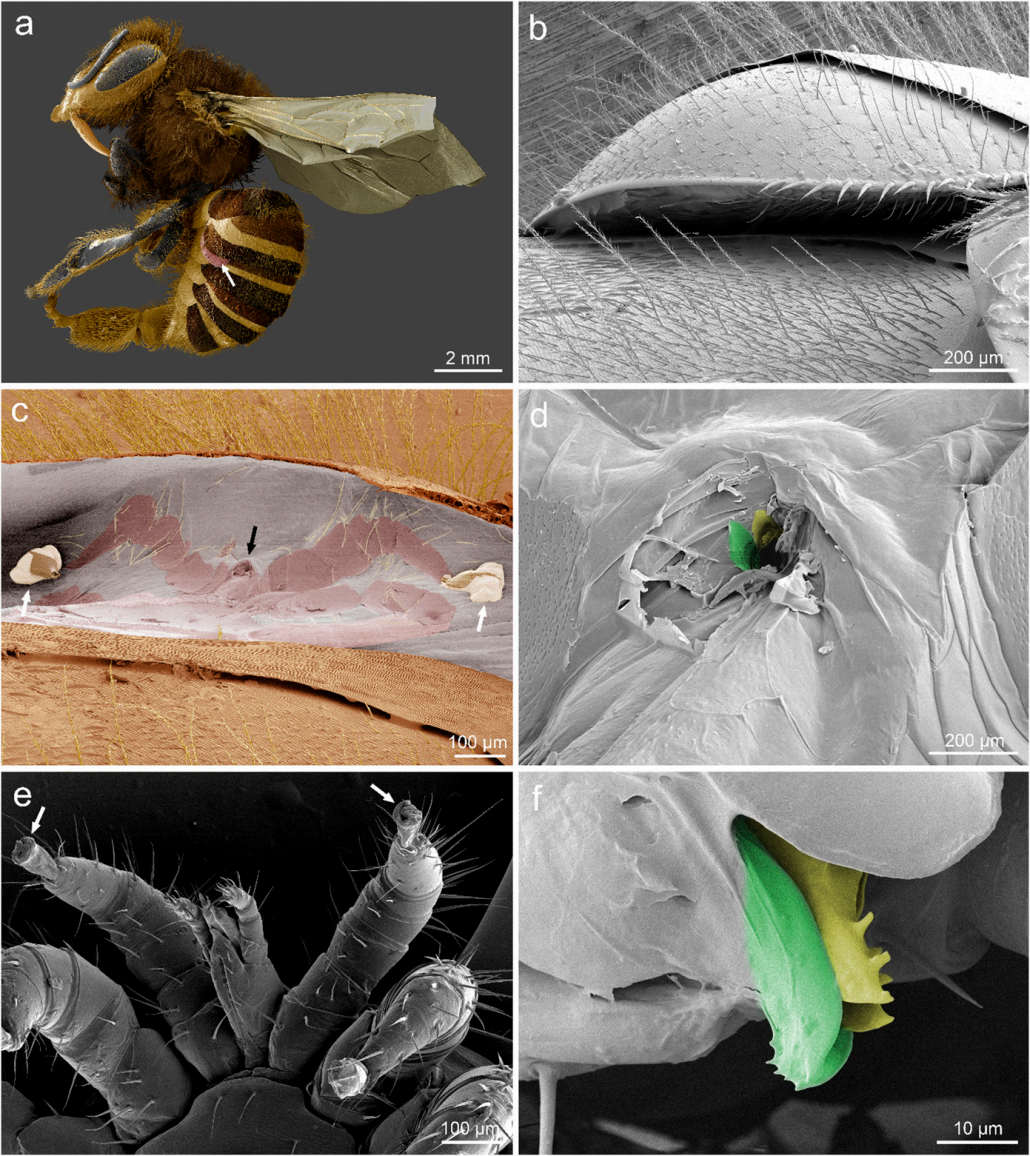
Feeding site of *Varroa* on adult honey bee imaged via low-temperature scanning electron microscopy. Images representative of 10 worker bees with attached mites prepared for imaging of which all 10 showed a wound in the intersegmental membrane. (*A*–*F*) Representative images of 10 bees parasitized by *Varroa*. Location of the mite shown with white arrow (*A*). The mite is wedged beneath the third tergite of the metasoma (*B*). When removed, a detailed impression of the mite can be observed in the intersegmental membrane in addition to a wound where the mouthparts of the mite would be (black arrow in *C*). Note, the ambulacra, or foot pads, of the mite (white arrows) remained attached to the membrane when the mite was extracted (*C* and *D*). Higher magnification of the wound reveals distinct grooves in the wound matching the modified chelicera of the mouthparts of the mite, colorized for clarity [moveable digit (yellow), corniculus (green)] (*F*). (*A* and *C*) Reproduced with permission from ref. 47.

We examined worker bees in the brood nests of four different colonies, of which 104 had at least one mite present. We observed distinct location biases in these mites (Fig. 1). The majority (*n* = 99, 95.2%) were found ventrally on the metasoma wedged underneath the overlapping terga or sterna (abdominal plates) of the bee (Figs. 1 and 2 *A* and *B*). Specifically, mites on the metasoma were found with greatest frequency (88.5%) underneath the sternite or tergite of the third metasomal segment (Fig. 1). No mites were found on the head of the host bee. Few mites were found on the mesosoma (thoracic region) of the host (about 4.8%). Notably, these mites behaved differently than the mites beneath the sterna and terga; they moved about actively and were primarily found with their sensory legs raised, suggesting that they were questing for transfer to a passing bee rather than feeding. Upon removal of the mite, we found no evidence of feeding in this location. Mites on the metasoma moved only after being disturbed repeatedly. *Varroa* located on the metasoma of the host also exhibited a consistent preference for the left side (74.8% of observations) (Fig. 1, *Left*).

The preference for feeding on the ventral rather than the dorsal region of the metasoma is consistent with expectations if fat body is the target tissue, as there are larger deposits of fat body tissue on the inner ventral surface of the metasoma rather than the dorsal surface. Preference for the third segment may be because it is the longest segment, providing a large external parasite with space to feed while concealing most of its body from a grooming host (Figs. 1, *Left*, and 2*A*). This theory is further bolstered by the observation that the mites consistently fed under the longest section of the longest segment, a lobe formed by the edge of each tergite or sternite (Fig. 2*B*). The strong preference of this parasite for the left side of its host suggests that there may be a benefit to feeding in this location as well. This preference may be related, in part, to asymmetry in host grooming habits or internal asymmetry of the host. Although the fat body does not show distinct differences in appearance based on where it arises in the metasoma, it may nonetheless have biochemical differences expressed in different regions of the tissue.

**Fig. 3.**
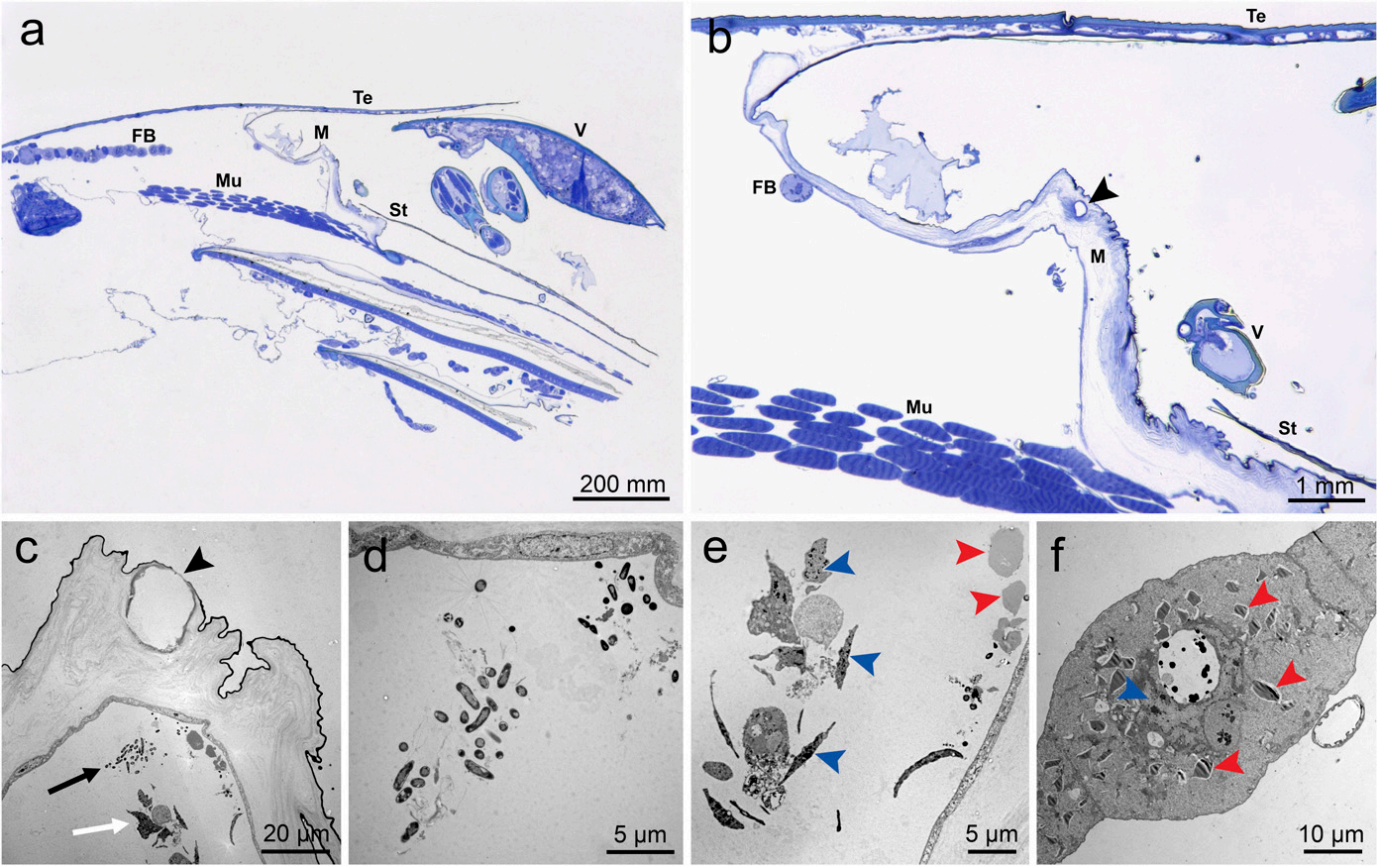
*Varroa* briefly parasitizing worker bees were used to pinpoint the precise location of the feeding site, revealing the ultrastructural morphology of the feeding wound, bacteria at the feeding site, and damage to the fat body after only hours of association with the host bee. Images captured via transmission electron microscopy. (*A* and *B*) Histological cross-section of a worker bee with *Varroa* attached between the third and fourth segments of the metasoma. Fat body tissue is shown beneath the intersegmental membrane (*A*). The wound caused by the feeding mite in the membrane of the bee is clearly visible as a large mound with a hole intersecting the membrane (arrowhead indicating the hole) (*B*). FB, fat body; M, intersegmental membrane; Mu, muscle tissue; St, sternite; Te, tergite; V, *Varroa*. (*C* and *D*) Feeding wound at higher magnification, showing a hole with irregular edges where mouthparts of the mite have penetrated the membrane (arrowhead). The black arrow indicates bacteria at the feeding site, and the white arrow indicates exposed contents of fat body cells likely due to the extraoral digestive processes of the feeding mite (*C*). Higher magnification reveals further detail distinguishing two morphologically distinct bacteria (*D*). (*E* and *F*) Higher magnification of degraded cell contents (*E*). Remnants of condensed chromatin from the nuclei of cells can be observed (blue arrowheads) in addition to the crystalline lipid droplets found abundantly in fat body trophocytes (red arrowheads) (*F*). (*A* and *C*) Reproduced with permission from ref. 47.

The life cycle of *Varroa* is separated into two distinct phases that focus on separate life stages of the bee: the reproductive (parasitizing the brood) and the phoretic (parasitizing adult bees). The term “phoretic” is defined by exploitation of a host exclusively for transport and specifically excludes exploitation of the host as a food source (39–46). To determine whether these regions of high activity are feeding sites or simply regions of the host that aid in phoresy, we examined the intersegmental membrane (membrane between segments of the metasoma) in the area of highest preference [as detailed in *SI Appendix*, *Extended Materials and Methods* and Ramsey et al. (47)]. Images captured via low-temperature scanning electron microscopy revealed a wound in the intersegmental membrane (Fig. 2 *C* and *D*) caused by the gnathosoma of the mite (Fig. 2 *E* and *F*). To better image the wound site, adult bees with apparently feeding mites were chemically fixed, thin-sectioned, and imaged via transmission electron microscopy. In addition, several 0.5-µm-thick sections were slide-mounted, stained, and imaged via light microscopy. These methods show a mound of host tissue at the wound site (Fig. 3 *A*–*C*). Just below the surface of the wound are the inner contents of damaged fat body cells showing degradation consistent with extraoral digestion (Fig. 3 *C* and *E*). Furthermore, these images also reveal two colonies of morphologically distinct bacteria (Fig. 3 *C* and *D*).

These images constitute direct evidence that *Varroa* feed on adult worker bees and are not using them for phoresy. Exceptions have been made historically for some parasites, such as *Macrocheles subbadius*, to still be considered phoretic, although they may or may not feed on their host briefly while in transit because transport to a specific destination is the primary goal, feeding is not consistent, and the parasite is not specialized for the task (40, 48, 49). This exception, however, cannot be made for *Varroa* because these mites utilize adult host bees for feeding consistently, their shape and anatomy is adapted for fitting between the plates of these bees to access their feeding site, and they remain in this phase for several days, showing that transport from one specific location to another is not the primary goal (50, 51). *Varroa* remain attached to adult bees between 1 and 13 d, with an average of about 7 d (50, 52, 53). If the primary goal of the mites is to be moved to a new reproductive host, the length of time spent on the adult host is unnecessary under most circumstances. *Varroa* are primarily found on nurse bees during this stage, and their frequent contact with viable brood would allow them to parasitize new brood cells almost immediately (54–56). This is the only portion of the lifecycle where the mites have the potential to be moved to other colonies, but were this the primary goal of this stage, one would expect that these mites would attach to foragers who leave the colony frequently rather than nurse bees, who rarely do so. These observations are, however, consistent with behaviors expected from a parasite with the goal of obtaining essential nutrients from a host.

These findings are also what is expected given those of Xie et al. (50), who determined that *Varroa* obtain a substantial fitness benefit from feeding on nurse bees but very little, if any, from newly emerged bees or foragers. The work of Xie et al. provided a reason for the observed preference of *Varroa* for nurse bees and our work provides further biological underpinning for that observation. The size and content of fat body tissue is not consistent over a bee’s life (37, 57–59). Both newly emerged bees and foragers have depleted fat body tissue (from the demands of metamorphosis in the former and changes associated with task shifting from feeding larvae in the latter), likely contributing to both life stages functioning as nutrient-poor host resources. Nurse bees have substantially larger and, ostensibly, more nutritionally dense fat body than other stages of the worker bee caste (60, 61).

The presence of bacteria in the feeding wound is a discovery of some concern because of recent studies detailing a connection between previously unrecognized bacteria and colony mortality (62, 63). A growing body of evidence has shown that even bacteria never known to be pathogenic can exhibit pathogenic characteristics under certain circumstances in honey bee colonies and, furthermore, work suggests an association of these infections with *Varroa* (62, 63).

**Fig. 4.**
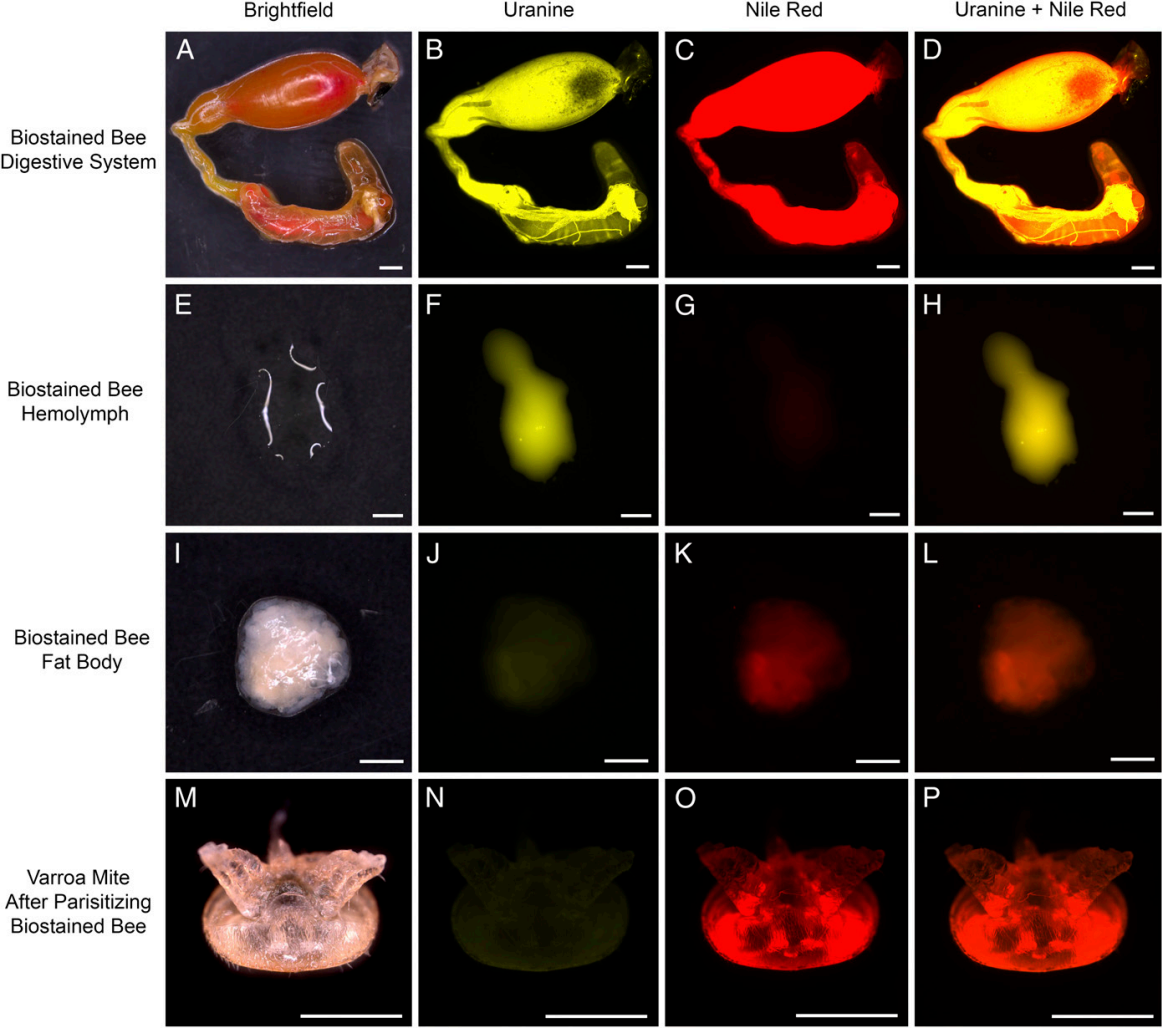
Host tissue collected from bees with fluorescently stained internal tissues showing the fidelity of each biostain, Nile red (lipophilic) and Uranine (hydrophilic), for each host tissue (fat body and hemolymph, respectively). *Varroa* are shown as well. Columns show honey bee tissue and a *Varroa* specimen fed on a biostained host bee in brightfield with successive columns showing fluorescence from these samples associated with Uranine, Nile red, and the two biostains imaged together. All scale bars represent 1 mm. (*A*–*D*) Gut tissue (*A*). Note: the high degree of fluorescence shown by both biostains (*B*–*D*). (*E–H*) Hemolymph tissue (*E*). Note: Uranine biostain shows high biochemical affinity for hemolymph (*F* and *H*). Barely discernible levels of Nile red (*G*) are likely a result of this biostain reacting to circulating lipophorin and cells present in the hemolymph. (*I*–*L*) Fat body tissue (*I*). Note: Nile red biostain shows high biochemical affinity for fat body (*K* and *L*). There are small amount of hemolymph present throughout fat body tissue that likely contributes to the low levels of Uranine fluorescence visible (*J*). (*M*–*P*) Photochemically cleared *Varroa* female (*M*). Note: very little fluorescence associated with the hemolymph can be seen (*N* and *P*); however, fat body fluorescence is intense with signal emanating primarily from the lobes of the digestive system (*O* and *P*).

While our observational and histological work provide evidence for fat body feeding, we set out to confirm our findings by differentially staining both target tissues in host worker bees and examining the contents of the mites allowed to feed on these bees. Bees were fed both a fluorescent lipophilic biostain, Nile red, to mark the fat body tissue and a fluorescent hydrophilic biostain, Uranine, to mark the hemolymph. Nile red was preferred to other lipophilic fluorophores because the intensity of red fluorescence of this fluorophore is substantially diminished or quenched altogether when immersed in polar fluids like hemolymph, which is primarily composed of water (64, 65). Samples of bee hemolymph, fat body, and gut were removed from the biostained host and imaged using fluorescence microscopy. These samples were used to verify that the correct biostain found and persisted in the target tissue (Fig. 4). Gut tissue was removed and imaged. As expected, a high concentration of both fluorescent biostains was clearly observed in the gut of the host (Fig. 4 *A*–*D*). When hemolymph was examined, the Uranine fluorophore showed high biochemical affinity for hemolymph and not the lipophilic Nile red. Barely discernible levels of Nile red are likely a result of this biostain reacting to low levels of lipophorin and circulating adipocytes (Fig. 4 *E*–*H*). Furthermore, the fat body exhibited the opposite of these qualities, sequestering Nile red and showing very little signal associated with Uranine. Fine-scale histology conducted on biostained host bees showed that even in the presence of high levels of Nile red, other tissues associated with the fat body (connective tissue, tracheoles, and so forth) absorbed little discernible fluorophore if any (*SI Appendix*, Fig. S3). A low volume of hemolymph present between the cells of the fat body likely contributed to the low levels of Uranine fluorescence visible in fat body tissue (Fig. 4 *I*–*L*). Bees with fat body or hemolymph that did not take up the target fluorophore were not used in the study. After feeding on host bees given both biostains, mites were photochemically cleared to suppress the competing autofluorescence of the exoskeleton of the mites and allow for imaging of internal fluorescence without dissection.

**Fig. 5.**
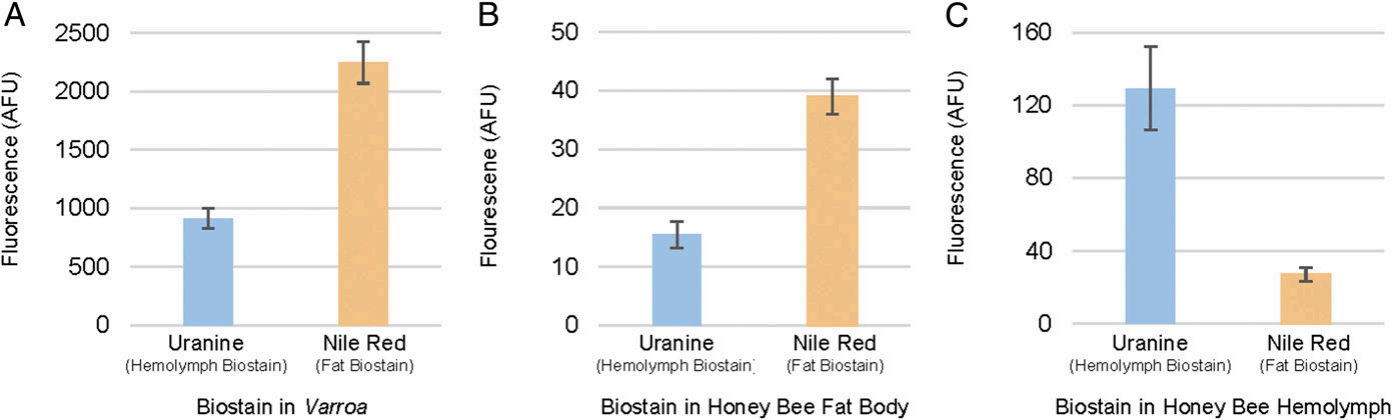
Mean fluorophore levels detected in *Varroa* and in biostained honey bee tissues. Fluorescence values reported in arbitrary fluorescence units (AFU). (*A*–*C*) Mean fluorophore levels detected in *Varroa* after 24 h of exposure to stained host bees (*A*). Levels of the fat body fluorophore (Nile red) are higher than that of the hemolymph (Uranine) fluorophore and appear in the same proportion as in the fat body of the host bees (*B*). Proportion test (prop test): χ^*2*^ = 3.62e-28, *P* = 1, *n* = 10. This proportion differs significantly from that of the hemolymph of these bees (*C*), providing further evidence that mites are not consuming this tissue in significant amounts (prop test: χ^*2*^ = 197.33, *P* < 0.001, *n* = 10).

A strong signal associated with the lipophilic biostain Nile red was consistently observed localized primarily to the rectum and multilobed gut of the mite, consistent with signal detected from the fat body tissue of the honey bee (Fig. 4 *M*–*P*). No mites from this study showed greater fluorescence levels from the hemolymph biostain than the fat body biostain. The distinct biochemical properties of each tissue allowed for a tissue-specific fluorescence profile to be determined. The proportion of fluorescence from the nontarget biostain relative to the target biostain was used to create the unique profile for each tissue. Nile red fluorescence over total sample fluorescence in honey bee fat body tissue yielded a value of 71.1% and 17.3% in the hemolymph (Fig. 5 *B* and *C*). Nile red fluorescence relative to total sample fluorescence generated from mites exposed to biostained bees yielded a value of 71.6% (Fig. 5*A*). There was no statistically significant difference between the fat body fluorescence profile and the fluorescence profile of the mites after feeding on biostained bees, providing further evidence that the tissue in the experimental mites is fat body tissue. In contrast, the fluorescence profile of the hemolymph (17.3%) differed substantially from what was found in the experimental mites.

**Fig. 6.**
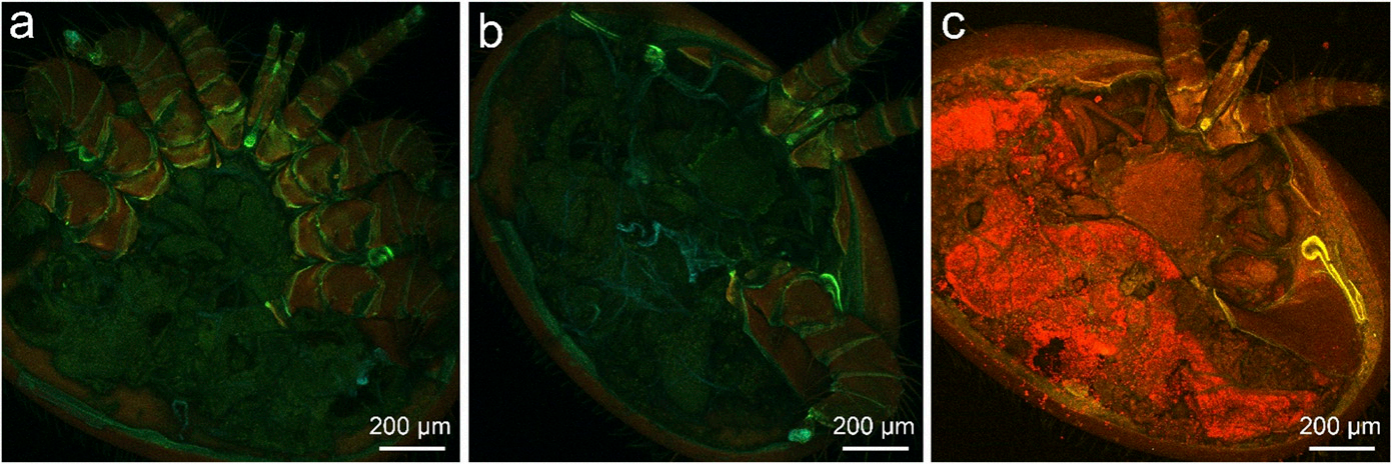
*Varroa* were fed on nurse bees given only one of the two fluorescent biostains and imaged via confocal laser scanning microscopy, verifying that low signal associated with hemolymph was not a result of the fat body fluorophore (Nile red) obscuring the fluorescence of the hemolymph fluorophore (Uranine). Mites that fed exclusively on bees with biostained hemolymph (*A*) showed fluorescence only marginally above the control (*B*). Mites fed on bees with fluorescently stained fat body show such high levels of Nile red in the digestive system that the shape of the gut can be clearly observed via fluorescence imaging (*C*). (*C*) Reproduced with permission from ref. 47.

To further validate the results of this study, an additional subset of bees was fed only one of the two fluorescent biostains to confirm that the consistently low signal recorded from the hemolymph biostain inside of the mites was not a result of the abundant Nile red fluorophore obscuring fluorescence from the Uranine fluorophore. Mites fed on bees given only a single biostain were imaged via confocal laser-scanning microscopy. To do so, we removed the genital and posterior metapodal plates (three plates on the posterior region of the venter covering the digestive and reproductive organs) from these mites to better resolve internal structures (Fig. 6). The confocal images allowed us to visually confirm our findings as mites fed on bees stained with only Uranine showed low levels of Uranine-associated fluorescence similar to the low values observed in mites fed on bees stained with both biostains (Fig. 6*A*). The gut and rectum of mites that had fed only on Uranine-stained bees was nearly indistinguishable from those tissues in the control mites visually, providing evidence that our protocols were not biased by the fat body fluorophore obscuring fluorescence from the hemolymph fluorophore (Fig. 6*B*). The images of the mites fed on bees with only stained fat body tissue further confirmed our overall findings as their gut tissue produced robust signal of sufficient intensity to clearly see the shape of the digestive system of the mites (Fig. 6*C*).

**Fig. 7.**
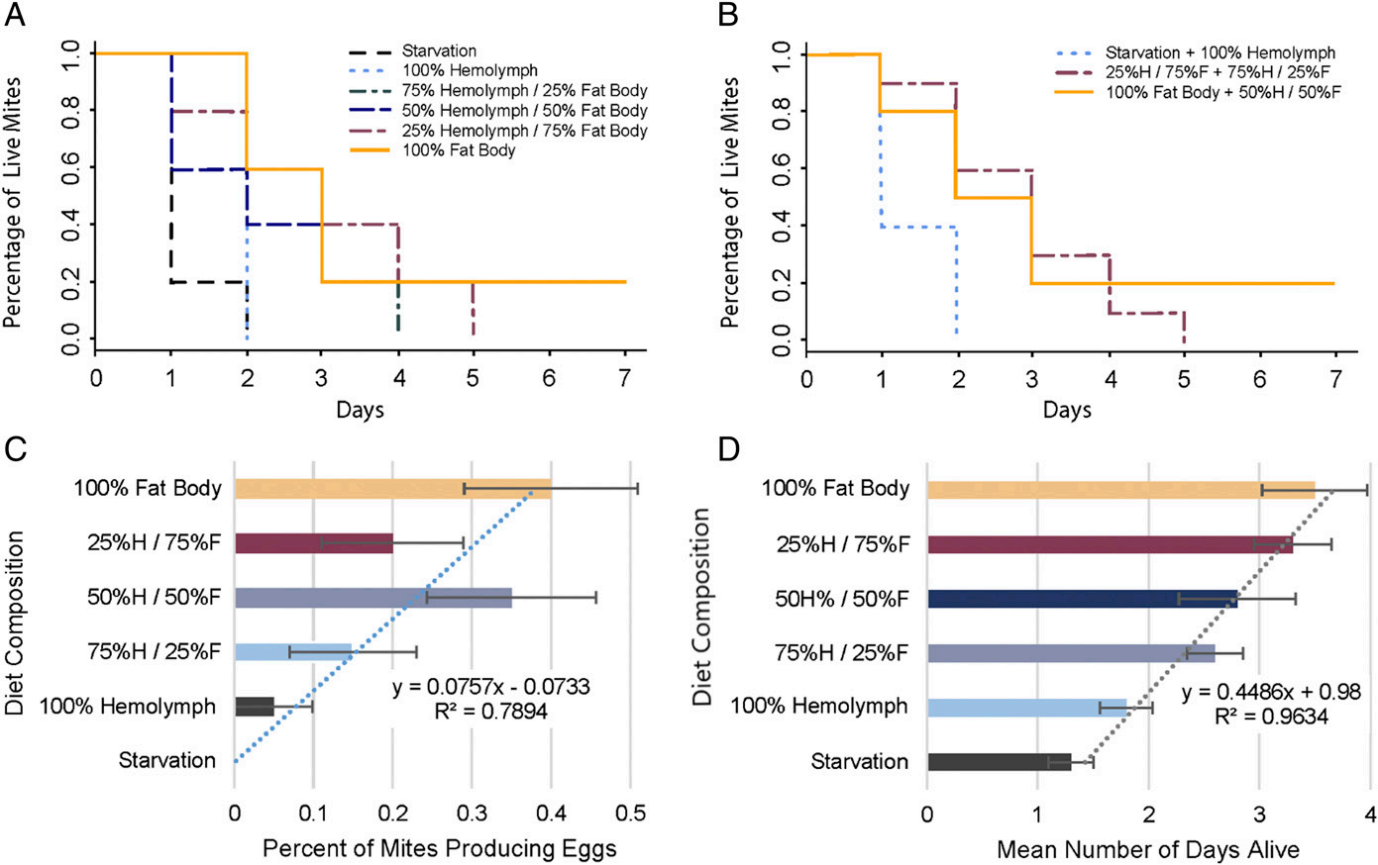
Mites fed honey bee fat body tissue survived longer and produced more eggs than mites provisioned with hemolymph. High mortality was observed across treatments, likely because of the artificial setting. After 3 d, mites receiving 0%:100% and 25%:75% hemolymph:fat body as their diet maintained survivorship at 60%, while the 100%:0% hemolymph:fat body and the starvation control had already exhibited full mortality. Final sample size consisted of 15 mites per treatment. (*A* and *B*) Survivorship curve showing starvation control and all five host tissue diets (*A*). (*B*) Representation of the same data with levels combined that show no difference in survivorship. Note: mites provisioned hemolymph and mites given no food showed no difference in survivorship. However, survivorship differed substantially between the hemolymph treatment and all treatments given any level of fat body (χ^*2*^ = 16.1, *P* < 0.001). (*C*) Egg production differed between treatment diets (ANOVA: *P* < 0.004). A positive linear relationship was observed between egg production and the amount of fat body in the diet of the mite (*R*^2^ = 0.7894). (*D*) Average survivorship of mites differed by diet. Survivorship and the ratio of fat body by volume adhere to a strong positive linear relationship (*R*^2^ = 0.9634).

Both studies provide evidence that *Varroa* consume fat body tissue when parasitizing adult honey bees. However, we also set out to determine if fat body is a dietary requirement impacting survival and fitness during the reproductive phase when *Varroa* feed only on honey bee brood. To answer this question, we developed a bioassay that provided reproducing *Varroa* in one of six host tissue diets with a hemolymph to fat body ratio of: 100%:0%, 75%:25%, 50%:50%, 25%:75%, 0%:100%, and an unfed control. We monitored mites provisioned with the different diets for 7 d, during which time we noted any oviposition and mortality. Mites that were provisioned with hemolymph only survived 1.8 ± 0.8 d on average, with 5% producing eggs that were not different from the group that was starved, living 1.3 ± 0.64 d with 0% fecundity (Fig. 7 *A*–*C*). As the concentration of fat body in the diet increased, survivorship and egg production increased as well (*r*^2^ = 0.9634) (Fig. 7*D*). Mites given no hemolymph, only fat body, showed the highest average rate of survivorship (3.5 ± 1.5 d) and fecundity (40%) compared to those fed all other diets (Fig. 7 *A*–*C*). Only mites in the 100% fat body or 50% fat body treatment survived the full 7-d duration of the experiment, albeit in relatively low numbers (20%). Mites provisioned with 100%, 75%, and 50% fat body in their diet had the three highest fecundity rates, respectively, at 40%, 20%, and 32%. The diet composed of 25%:75% hemolymph:fat body contributed far more rapidly to the growth of fungus than the other diets and, as such, a disproportionate number of experimental units were lost, likely contributing to the lower-than-expected survivorship and fecundity in this treatment.

The rate of egg production for mites on our most successful diet, 100% fat body (40% fecundity), was on the lower end of fertility rates documented in natural conditions (varying between 40% and 80% in worker brood based on factors that have not yet been fully determined) (1, 66). The relatively low fecundity rates and low survivorship are likely explained by the artificial nature of this laboratory study. In addition, our decision to avoid including additives that may change the palatability of the mite’s diet (such as antifungals) periodically contributed to the growth of fungi and other microbes that created a constant challenge in the rearing process. Artificial rearing of *Varroa* off host is still a challenge with no recognized rearing protocols available thus far (1, 67). However, the consistency of our methods across treatments and stark differences in outcomes between treatments still allowed for the recognition of clear trends.

## Conclusions

These findings provide sufficient evidence to reject the conventional model that *Varroa* are hemolymphagous parasites. The location of their feeding site, the predigested fat body cells therein, the presence of lipid-dense host tissue in the gut of the mite, and the strong relationship between survivorship, fecundity, and the levels of fat body in the diet of the mite all suggest that the primary host tissue consumed by *Varroa* is the fat body. This fundamentally changes our understanding of this agriculturally significant parasite and has important implications for bee researchers attempting to understand the etiology of varroosis. Detailed imaging of the feeding site provides direct evidence that the stage parasitizing adult bees is not a nonfeeding phase, as the name presents but, in keeping with the body of evidence from several other studies, is a portion of the lifecycle where feeding is a goal for which this parasite is uniquely specialized (50, 51, 68, 69). These findings emphasize a need to revisit how we discuss the lifecycle of this parasite.

The development of tools, both chemical and nonchemical, to manage this pest is particularly likely to be affected by these findings. The in vitro feeding system used in this study maintains *Varroa* off-host for more than a week. With further refining of culturing conditions (e.g., cell design, ventilation, and so forth), it may be possible to rear mites for the full 12- to 14-d period of their reproductive cycle in capped cells, contributing useful insight into the intricacies of and potential vulnerabilities in their lifecycle. These findings further help to explain why past attempts to develop in vitro *Varroa*-rearing models have failed (67, 70), because they attempted to use diets based on the dilute nutritional content of honey bee hemolymph rather than fat body tissue. Similarly, lack of success in developing effective systemic pesticides likely is because of the same issue of tissue misidentification (24). It was considered fact at the time of their development that mites fed on hemolymph; thus, these pesticides were likely formulated to persist in the hemolymph of the honey bee rather than the fat (24). These findings have practical implications for the development of novel *Varroa* management technologies, such as systemic interfering RNA, which would need to be formulated to accumulate in fat tissue to target this parasite.

These results mark an advancement in our understanding of exactly how *Varroa* feeding impacts honey bees. *Varroa* parasitism is associated with impaired development of immature bees (5), decreased lipid synthesis (5), reduced protein titers (5), desiccation (5, 6, 8), impaired metabolic function (5, 9), inability to replace lost protein (9), precocious foraging (58), heightened winter mortality (57), impaired immune function (10, 11, 27), decreased longevity (24, 57), and reduced pesticide tolerance (12, 71, 72) (*SI Appendix*, Fig. S5). This diverse array of pathologies was difficult to account for under the conclusion that the parasite is feeding on hemolymph but is well-explained by exploitation of the multifaceted fat body tissue. For example, the removal of hemolymph has never been sufficient to explain why bees fed on by *Varroa* are unable to replace lost protein, show impaired ability to synthesize lipids, have diminished immune capacity, or why adult bees fed on as brood lose a large volume of water leading to desiccation. Hemolymph is not a storage site for protein, does not synthesize lipids or antimicrobial peptides, and is not removed in levels sufficient to cause desiccation (5, 73, 74). However, the role of the fat body as the primary storage and synthesis site for protein and lipids would explain why adult bees parasitized by *Varroa* as brood are unable to store protein from the pollen consumed in their diet as adults and why the synthesis of fat is inhibited (35, 73, 74). It would further be expected that substantially damaged fat body tissue would be hampered in its ability to produce antimicrobial peptides, lipophorin, and wax precursors, with the former being critical in immune response and the latter two in maintaining the water-proof seal around the body, which prevents the evaporation of water and subsequent desiccation. The fat body further facilitates metamorphosis, regulates metabolism, and plays an integral role in thermoregulation (35, 73, 74).

The role of the fat body in protein synthesis may also account for early task shifting as the fat body produces vitellogenins, which are essential in signaling task shifting in addition to the roles it plays in immune function and reduction of oxidative stress (35, 58, 75). Our findings, like those of Kuster et al. (76) and Annoscia et al. (27), support the conclusion that simply the removal of tissue in pupae is enough to diminish the immune response of the bee, in contrast to previously proposed means of direct immunosuppression (10, 37). While the tissue in these studies was referred to loosely as hemolymph, hemolymph removed at this stage would invariably include a large volume of fat body, contributing to the depletion of both immune factors and the tissue tasked with producing more of them (35, 37). The impact of *Varroa* on multiple facets of the honey bee’s immune response (reduction in vitellogenin titers and antimicrobial peptide production) is of special concern because of the constantly expanding complex of microorganisms associated with this parasite (1, 2, 62, 63). Further work should be conducted to identify the two morphologically distinct bacteria observed at the wound site, as previously nonpathogenic bacteria have recently shown capacity for pathogenicity, with more than 90% of bees in some failing colonies having a pathogenic strain of *Serratia marcescens* (62).

Evidence of extraoral digestion in this study provides further weight to the finding that a significant volume of apparent salivary content is left behind after *Varroa* feed (5). How long this material remains bioactive is not yet known, but likely extends the impact of feeding beyond the volume of tissue directly consumed by the mite. In addition, when parasitizing brood, *Varroa* feeding events are frequent and result in the removal of about 0.86 µL of tissue after 1.5 h (24, 77). Similar behavior during the average of 7 d that *Varroa* spend parasitizing adult bees would likely lead to substantial damage to fat body tissue after only a few days (50, 52, 53). These implications are relevant to natural and induced broodless periods that force the entire population of mites onto adult bees, where their feeding damages essential tissue and transmits viruses.

Fat body tissue also plays a crucial role in pesticide detoxification by absorbing and sequestering a wide range of xenobiotics, thereby preventing them from finding their active site and causing damage (74, 78). Recent work has shown that honey bees fed upon by *Varroa* suffer damage from pesticides even at concentrations that previously would have been inert, suggesting that their feeding on this tissue may disrupt the process of pesticide detoxification (71). This factor potentially plays a role in the observed honey bee health decline, considering the near ubiquitous presence of *Varroa* in honey bee colonies and the heavy reliance globally on chemical pesticides. Exploitation of this pathway as a miticide delivery strategy may be possible if a miticide tolerable to the bees can be incorporated into the bees’ feed to be subsequently absorbed by the fat body during digestion and delivered to the mites when they consume this tissue.

Healthy fat body tissue is also critical to overwintering success; thus, these findings underscore an imperative for beekeepers to reduce *Varroa* populations in colonies before the emergence of so-called “winter bees.” Simple reduction of mite loads late in the season to decrease the overwinter parasite load may not be enough, as it still allows for the mites to damage tissue critical to the process of overwintering as the bees prepare for this period. Vitellogenin produced by and stored in the fat body reduces oxidative stress, substantially extending the lifespan of the bees during the winter (57, 58). Impairment of this function is expected to adversely impact winter survival and spring build-up. Removal of fat body tissue from bees developing below the capping would also likely interfere with the process of metamorphosis. Fat body is integral to the success of this process. Because enzymes produced by the immature bee work to disintegrate its larval organs, those macromolecular components are absorbed by fat body dispersed throughout the body to be slowly released during the pupal stage to structure the adult organs (37). Removal of fat body tissue during this critical process would ostensibly have implications for the eventual size and health of the adult insect. A treatment schedule that includes treatment in late summer or early fall before mites can significantly damage fat body in developing winter bees would likely be more effective. The ability of this parasite to negatively affect such a broad array of processes further highlights the pivotal link between this parasite and honey bee health. Our study reflects a need to reexamine even the fundamentals of our knowledge of *Varroa* as we work to diminish its impact.

## Materials and Methods

### Spatial Distribution of *Varroa* on Worker Bees.

To determine the location of *Varroa* on adult bees in *A. mellifera* colonies, we examined bees originating from naturally mite-infested colonies. Between May and June 2016, frames containing capped and uncapped brood were removed from four colonies on eight occasions. Immediately after removal, worker bees were randomly selected, pulled from the frame by clasping the wings together, and inspected for the presence of *Varroa*. The location of the mite was recorded being on the head, between the head and mesosoma, on the mesosoma, between the mesosoma and metasoma, or beneath an ordinal numbered tergite or sternite on the metasoma (24 locations total).

### Tissue Biostain.

Approximately 30 newly emerged worker bees were confined to cages. Bees were allowed to feed ad libitum on a 30% sucrose solution containing both a lipophilic fluorescent biostain to mark the fat body, Nile red (Thermofisher), and hydrophilic biostain to mark the hemolymph, Uranine (Thermofisher). These biostains were also introduced in an artificial pollen substitute (Megabee). At 5 d posteclosion, biostain-fed bees were removed from colony cages with the other bees and placed singly in a small transparent 1.25-oz Solo cup with nylon mosquito netting used as a lid to ensure that the mites were not able to escape. A single *Varroa* female was placed on the body of each adult bee. After the trial, the *Varroa* were removed from their host bees. Autofluorescence of the integument was quenched by submerging the mites in 30% hydrogen peroxide, allowing for fluorescence imaging to occur through the integument without dissection.

### Tissue Feeding Bioassay.

*Varroa* for this study were collected directly from the sealed brood cells. This length of time proved important as the mites appear to react to environmental cues that potentially induce transition into their reproductive phase during this period. In preliminary trials, mites removed from cells before 12 h produced very few if any offspring, regardless of treatment. Reproductive mites were transferred to artificial enclosures and given 20 µL of honey bee tissue through an artificial membrane. This membrane was composed of parafilm stretched to about 15 µm in thinness. Five foundress mites were randomly assigned to each treatment per trial and three trials were conducted. Treatment solution consisted of one of the following formulations: 75% hemolymph to 25% fat body, 25% hemolymph to 75% fat body, 50% hemolymph to 50% fat body by volume, 100% hemolymph, or 100% fat body. Survivorship was recorded once per day over the course of 7 d.

## Supplementary Material

Supplementary File
