# Supplementary material for: Varroa destructor feeds primarily on honey bee fat body tissue and not hemolymph
Source: Proc Natl Acad Sci U S A. 2019 Jan 15;116(5):1792–801. doi: 10.1073/pnas.1818371116 (PMC6358713; doi:10.1073/pnas.1818371116)
Supplement: Supplementary File [file pnas.1818371116.sapp.pdf]

PNAS
www.pnas.org

Supplementary Information for

Varroa destructor feeds primarily on honey bee fat body tissue and not hemolymph

Samuel D. Ramsey[*], Ronald Ochoa, Gary Bauchan, Connor Gulbronson, Joseph Mowery, Allen Cohen, David Lim, Judith Joklik, Joseph M. Cicero, James D. Ellis, David Hawthorne, Dennis vanEngelsdorp

*corresponding author: (240) 393-6011, sramsey2@umd.edu,

This PDF file includes:

Figs. S1 to S5
Materials & Methods
Statistical Analysis

www.pnas.org/cgi/doi/10.1073/pnas.1818371116

Supplementary Figures

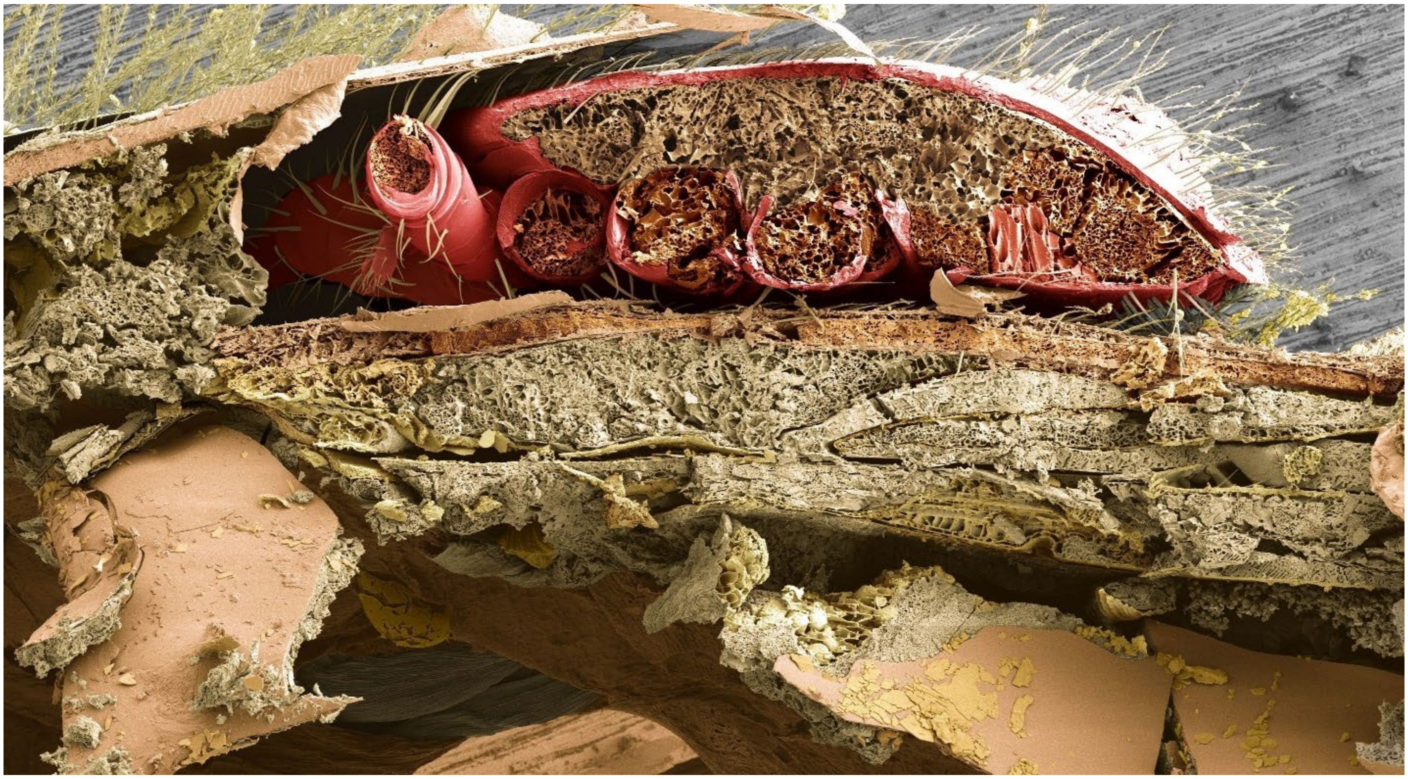

Fig. S1. Low temperature scanning electron microscopy freeze fracture image showing a cross section of a mite at the feeding site located between the overlapping plates of the metasoma (abdominal region) of the host bee.
Reproduced with permission from ref. 4.

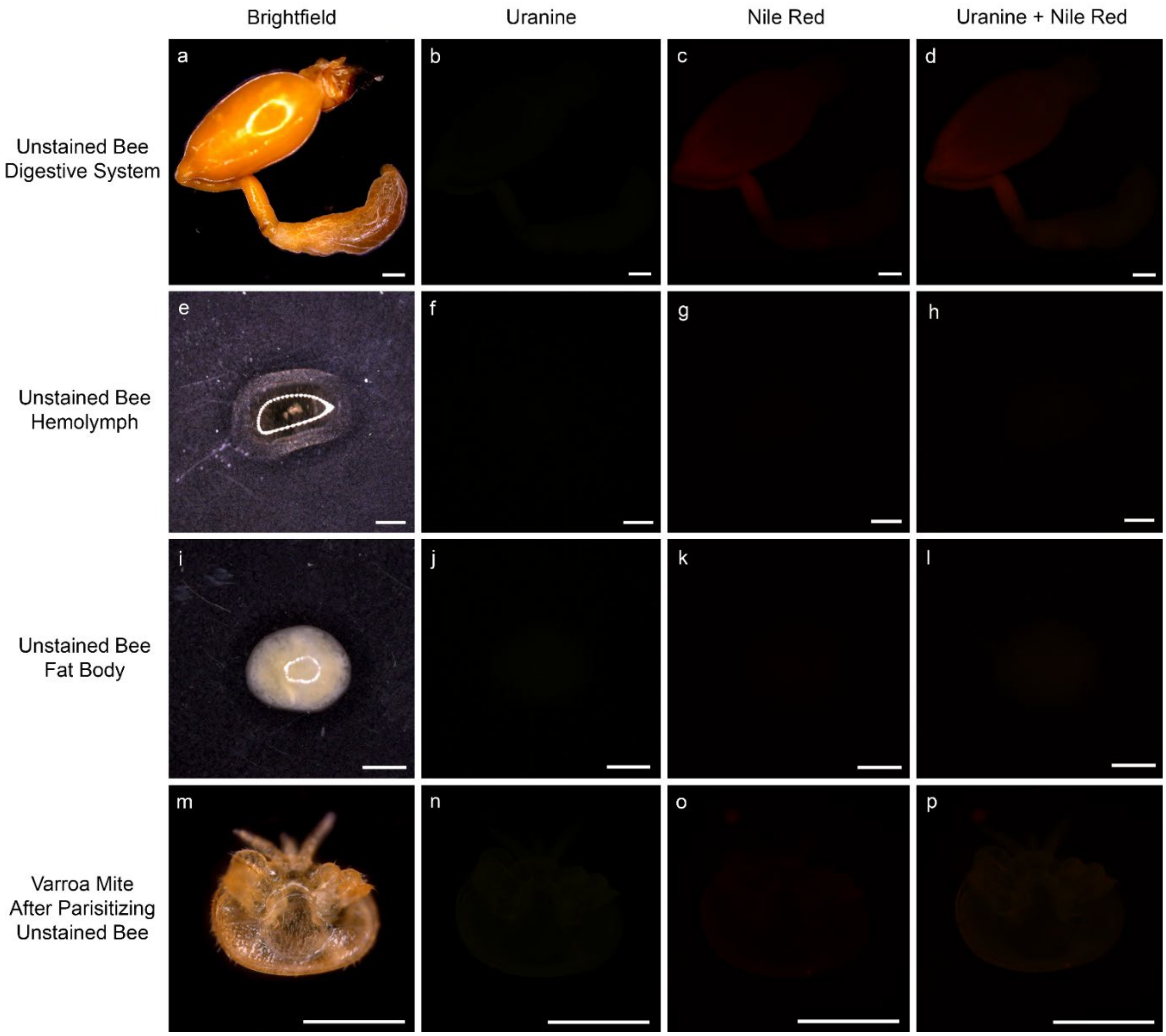

Fig. S2. Control for the localization of fluorescent biostains in honey bee tissue and inside *Varroa.* Host tissue was collected from bees given diet with no biostain (sugar water only). Autofluorescence was consistently low across honey bee tissue samples and within the photochemically cleared mites fed on the control bees. Rows show honey bee tissue and a *Varroa* specimen allowed to feed on these bees. Columns show fluorescence emissions in the yellow field (associated with Uranine), the red (associated with Nile Red), and the two together. All scale bars represent 1mm.

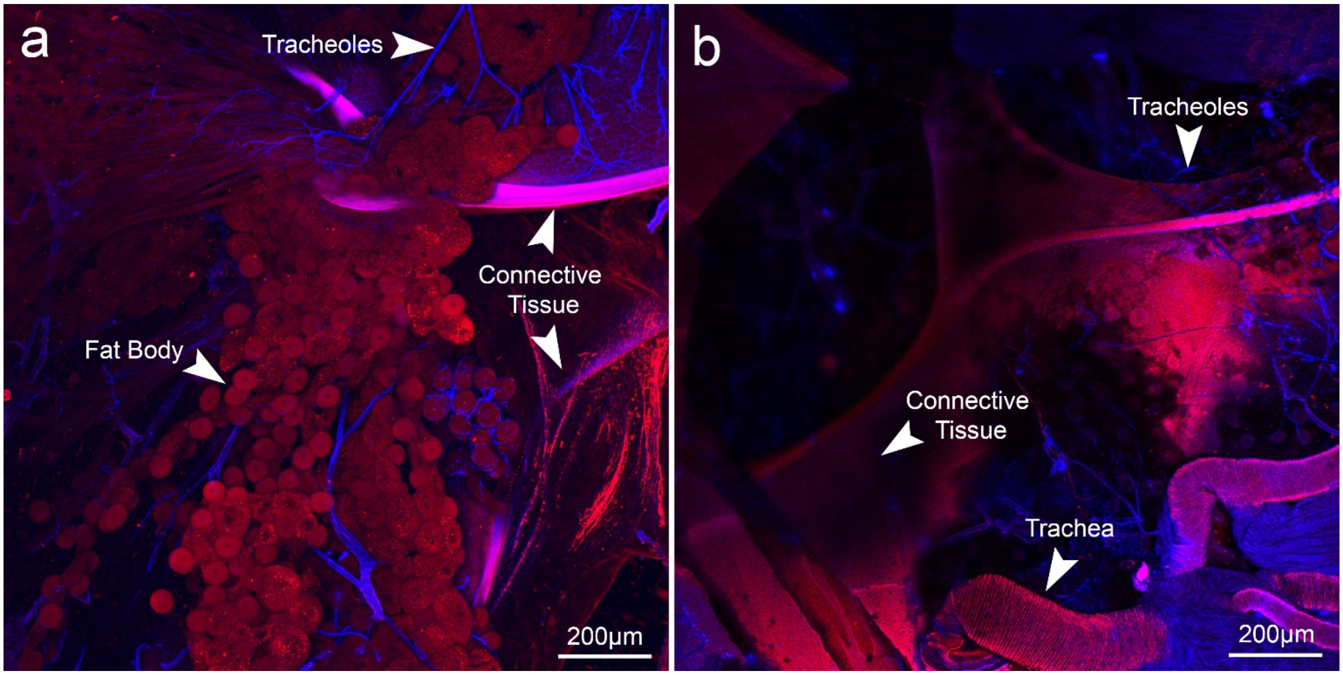

Fig. S3. Confocal laser scanning microscopy images of fat body tissue in 5-day old nurse bees having consumed Nile Red biostain (**a.**) or no biostain (**b.**) Fluorescence associated with faintly fluorescent red tissue is a result of the natural autofluorescence of these tissues (connective tissue and the taenidia of unbranched tracheal trunks). In image **a**, trophocytes and oenocytes, the primary constituents of honey bee fat body tissue, can be observed stained red with tracheoles (blue) infiltrating these cells to facilitate gas exchange. Fat body tissue is not clearly apparent in the dark regions of image **b**, as it emits very little autofluorescence. Tracheoles in this image (**b.**) appear to arise without terminating in a tissue likely because the fat body cells that they aerate are not visible without the biostain. Note: The consistent autofluorescence of the connective tissue, trachea, and tracheoles is unchanged between the fluorescently stained treatment group and the control further evidence of the biochemical specificity of Nile Red for lipid dense tissues.

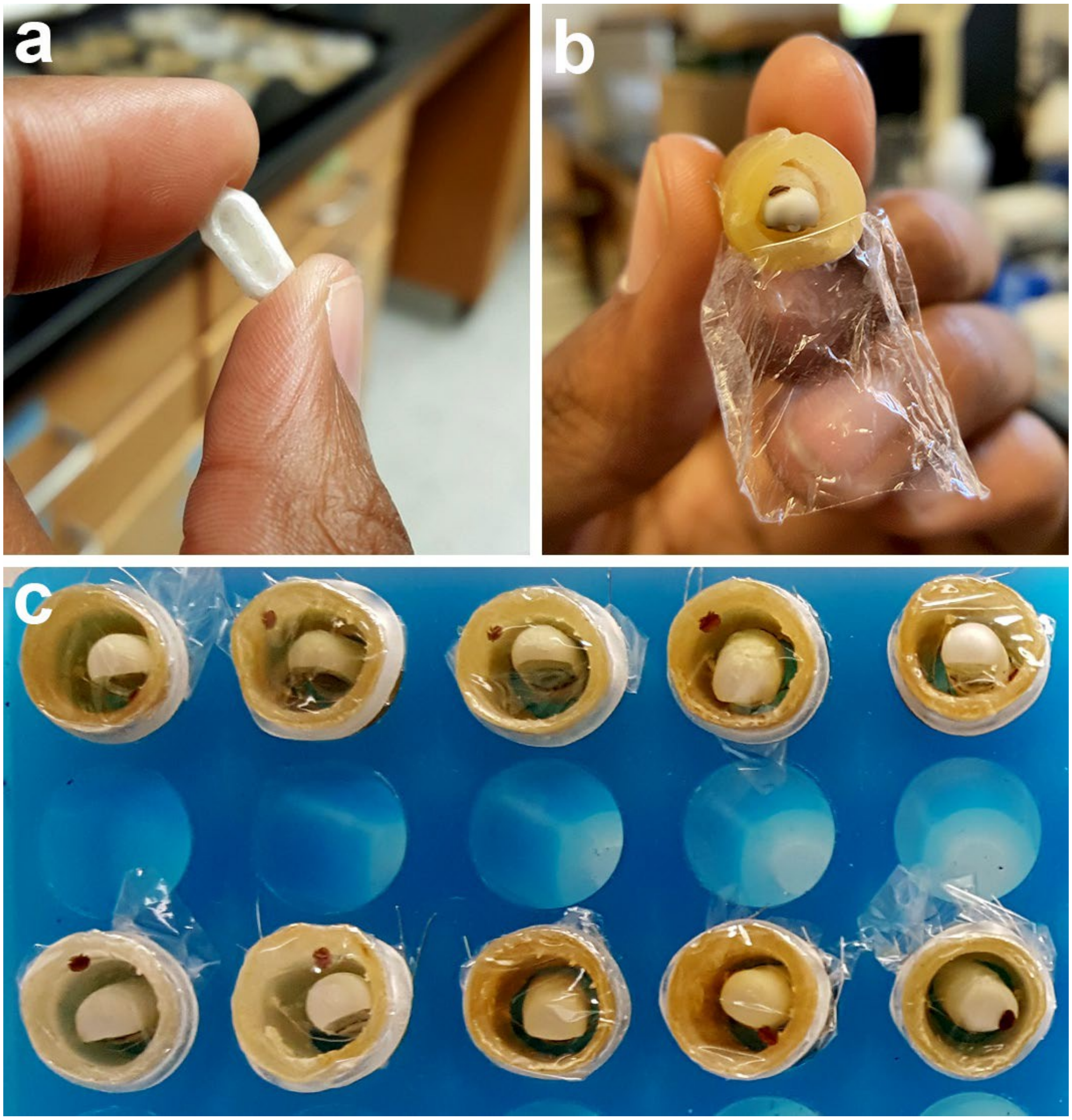

Fig. S4. In vitro *Varroa* mite rearing system used to feed mites a controlled diet of specific honey bee tissues. Decoy brood was composed of a gelatin capsule with a section cut away and replaced with a layer of parafilm stretched to 15µm in thinness (**a, b**). Enclosure made of compressed bees wax with transparent clingwrap used as cell capping (**b, c**).

Fat Body Function	Associated Pathologies Reported in Parasitized *A. mellifera*
**Regulation of Growth & Development/ Facilitation of Metamorphosis** *(Amdam et al. 2003; Amdam et al. 2004; Mirth et al. 2007; Arrese & Soulages 2010; Stell 2012)*	**Stunted growth, malformed organs, precocious foraging** *(Bowen-Walker and Gunn 2001; Amdam et al. 2004; Nilsen et al. 2011; Rosenkrantz et al. 2010)*
**Nutrient Storage and Mobilization** *(Keeley 1985; Arrese & Soulages 2010)*	**Inability to replace and store amino acids; reduction in amino acid and carbohydrate levels** *(Bowen-Walker 2001; Doormalen 2013)*
**Pesticide Detoxification** *(Locke 1980; Landa et al. 1991)*	**Increased susceptibility to pesticides** *(Gregorc et al. 2012; Blanken et al. 2015)*
**Osmoregulation** *(Keeley 1985; Arrese & Soulages 2010)*	**Increased water loss and associated weight loss** *(Bowen-Walker & Gunn 2001; Salvy et al. 2001; Annoscia et al. 2012)*
**Production of Antimicrobial Peptides** *(Amdam et al. 2003; Arrese & Soulages 2010)*	**Diminished immune response** *(Yang 2005; Yang 2007)*
**Thermoregulation** *(Locke 1980; Arrese & Soulages 2010)*	**Greater overwinter mortality** *(Sammataro & Needham 1996; Bowen-Walker & Gunn 1998; Amdam et al. 2004)*
**Regulation of Metabolic Activity** *(Locke 1980; Keeley 1985; Arrese & Soulages 2010)*	**Reduction in oxidative phosphorylation & overall metabolic rate** *(Bowen-Walker & Gunn 2001; vanDoormalen 2013)*
**Protein & Lipid Synthesis** *(Locke 1980; Keeley 1985; Arrese & Soulages 2010)*	**Decrease in lipid and amino acid production** *(Tewarson 1983; Glinski & Jarosz 1984; Weinberg & Madel 1985; Bowen-Walker & Gunn 2001; Amdam et al. 2004; vanDoormalen et al. 2013)*
**Vitellogenesis** *(Amdam et al. 2003; Arrese 2010; Nilsen et al. 2011)*	**Reduction in vitellogenin titers; decreased lifespan; increased overwinter mortality** *(Tewarson 1983; Amdam et al. 2003; Amdam et al. 2004)*

Fig. S5. Detailing the primary functions of the fat body in honey bees and pathologies related to these functions that result from *Varroa* parasitism. Note: Each function of the fat body is adversely impacted by *Varroa* parasitism.

-Extended Materials & Methods:

Spatial Distribution of Apparently Feeding Mites on Adult Bees

To determine the location of *Varroa* on adult bees in *Apis mellifera* colonies, we examined bees originating from naturally mite infested colonies maintained by the University of Maryland or United States Department of Agriculture, Agricultural Research Service (USDA ARS) Bee Research Lab in Prince Georges County, Maryland. Between May and June 2016, frames containing capped and uncapped brood were removed from 4 different colonies on 8 occasions. Sampling was conducted during favorable beekeeping weather (i.e. while sunny and warm) to permit opening hives with minimal usage of smoke, minimizing the potential impact on parasite behavior. Immediately after removal, worker bees were randomly selected, pulled from the frame by clasping the wings together and inspected for the presence of *Varroa*. The location of the mite was recorded being on the head, between the head and mesosoma, on the mesosoma, between the mesosoma and metasoma, or beneath an ordinal numbered tergite or sternite on the metasoma (24 locations total). Examined bees were removed from the colony to avoid sampling the same individual multiple times. Data from drones was not collected in this study.

Tissue Biostain

Frames of capped brood were collected from untreated colonies maintained by the University of Maryland, College Park. Each individual frame of capped brood was placed in a ventilated, single frame box (custom made by Zastrow Services LLC) and placed an incubator at 34°C and 80% RH. Emerging adult bees were collected at least once every 24 hours and the mites allowed to remain on the host bees until needed later in the study.

Adult honey bees were confined to cages with approximately 30 individuals of the same age. Cages consisted of 16oz transparent *Solo* brand cups with nylon mosquito netting used as a lid to maintain adequate ventilation. Feeders were made from 2ml microcentrifuge tubes. A hole was made in

the bottom of each feeder with a heated metal probe. Four feeders were suspended through small holes cut in the mesh lid of each cage.

Fluorescent biostains were used to stain the relevant tissues. Several biostains including Oil Red O, Rhodamine B, and Sudan III were evaluated. Uranine (*Thermofisher*), was selected to mark the hemolymph because of its low lipophilicity (k_{ow}: 0.0342) and high water solubility (500,000 mg/L). Nile Red (*Thermofisher*) has the inverse of these characteristics (k_{ow}: 4.38, solubility: 0.178 mg/L) and as such was used as the fluorophore to mark the fat body. Nile Red was preferred to other lipophilic biostains as its unique biochemical properties contribute to this biostain having substantially diminished fluorescence when in polar substances like hemolymph which is primarily water. Thus, if low levels of Nile Red make it into the fluid hemolymph (dissociated from lipophorin or cells in the hemolymph) the biostain would be quenched.

The diet consisted of 30g of sucrose dissolved in 100ml of distilled water. A 2% mixture of Uranine was made from 2g of sodium fluorescein powder dissolved in 98ml of distilled water. Then 1.5ml of the 2% Uranine mixture was added to the sugar water diet. A mixture of 0.025g of Nile Red biostain powder dissolved in 0.5ml of sunflower oil was added to the solution. Soy lecithin (0.5g) was added to act as an emulsifier and 5g of honey as a stabilizer for the emulsion. The entire mixture was blended for 1 minute in a high speed *Nutribullet* blender (*Pro 900 Model*) to ensure thorough mixing of the emulsion. Honey bees were allowed to feed on this solution *ad libitum* immediately post eclosion for 5 days.

We also introduced *Megabee* pollen substitute as a source of protein. Burr comb was removed from several honey bee colonies and placed into the aforementioned rearing cages. We created a 100ml, 1:1 mixture of pollen substitute to heavy sugar syrup (composed of 2:1 sugar to distilled water). We then added 5ml of sunflower oil mixed with 0.025g of Nile Red powder and 1.5ml of the 2% Uranine mixture. This pollen/fluorophore mix was added to about 20 cells per comb.

At 5 days post eclosion, biostain-fed bees were removed from colony cages with the other bees and placed singly in a small transparent 1.25oz *Solo* cup with nylon mosquito netting used as a lid to ensure that the mites were not able to escape. A single *Varroa* female was placed on the body of each adult bee. The bee was allowed to feed on 30% sugar solution *ad libitum* from a partially filled 2ml microcentrifuge tube suspended through a hole in the mesh lid. This sugar solution lacked biostain to prevent mites from picking up biostain from the feeder or potential spills while confined with the experimental host bee. Bees in our cage studies showed far greater survivorship when confined with burr comb so small sections of burr comb (~9 cells) were cut out and placed in these small cups as well. Pollen mix without either biostain was added to 4 cells.

After the trial, the *Varroa* were removed from their host bees. The mites were then rinsed with 70% ethanol to remove biostain that may have potentially contacted the mite's integument from the excrement or regurgitation of the host. The experimental mites were then placed in fluid exchange vessels, 1 mite per numbered well to allow for continued association of the mite data with data from its specific host bee. These fluid exchange vessels were then submerged in 30% peroxide for 5 days to quench the autofluorescence of the integument of the mite. After the peroxide bath, fluorescence levels of the internal structures were acquired and quantified using a *Zeiss Axiozoom V16* stereo zoom microscope. Images of the mites were captured at 40x magnification at 1s exposure. Fluorescence values were aggregated using ImageJ software and analyzed with R statistical computing software version 3.4.2.

Tissue was extracted from each experimental bee to verify that the correct biostain accumulated in the target tissue. Using a *Finnpipette* brand 10µl micropipette, 2µl of hemolymph were withdrawn from each bee. Afterwards, the gut contents were removed from the bee by carefully pulling out the digestive tract via the stinger. If the digestive tract showed any signs of tearing, that sample was discarded to avoid gut contents spilling onto the fat body and causing inaccurate readings. Fat body was

collected via dissection and about 2µl was measured using a *Finnpipette* brand 10µl micropipette. If hemolymph or fat body tissue samples from a bee showed fluorescence levels lower than 1 standard deviation from the mean value detected in the overall population of bees fed these biostains for 5 days, the bee and associated mite were not used in this study. Fluorescence of honey bee samples was acquired using the same methods as those for *Varroa*. Samples were also collected via the same methods from honey bees fed sugar solution lacking any fluorophore. The mites fed on control bees submerged in 30% hydrogen peroxide and imaged to establish baseline autofluorescence levels for photochemically quenched mites. Tissue from the control bees was used to estimate average levels of autofluorescence naturally associated with each tissue. The averages of these values were calculated and removed from those of the experimental mites prior to statistical analysis.

Our preliminary observations showed that *Varroa* rarely survived for 24 hours without feeding which corresponds with the findings of Garedew et al. (2004)(1). To reduce the likelihood that data from *Varroa* that had not fed on the biostain marked host were being included in this study, *Varroa* that died during the 24-hour trial were not processed or included in the data set. Trials in which the honey bee did not survive were also not included. Though *Varroa* have been shown to continue feeding from bees several hours after they die (1), hemolymph samples could not be obtained from dead bees so their data was also not included in the analysis.

Preliminary trials were conducted to establish the stability of each biostain in the target and non-target tissue. Adult bees were randomly assigned to one of two treatments, each fed only one of the biostains from the first day of emergence onward. After 5 days the levels of the biostain in hemolymph and fat body were collected and compared using the aforementioned protocols. Peroxide protocols were refined to ensure this chemical was not damaging the fluorophore. Mites fed biostain were submerged in peroxide for between 1 & 12 days to determine when autofluorescence was adequately quenched but the fluorophores were still active. This period was determined to be between

4 and 6 days.

Tissue Feeding Bioassay

Varroa for this study were collected directly from the sealed brood cells of heavily infested colonies maintained by the University of Maryland, College Park or the USDA ARS. Cells were selected that had been capped for approximately 12 hours. This length of time proved important as the mites appear to react to environmental cues that potentially induce transition into their reproductive phase during this period. In preliminary trials, mites removed prior to 12 hours produced very few offspring regardless of treatment. Significantly longer than 12 hours and the mite is likely to begin feeding on the developing prepupa. The end of this period is marked by the initiation of the cocoon spinning phase thus larval age could be estimated accurately based on the presence of silken fibers lining the underside of the capping. The results of this study could potentially be biased if some *Varroa* were able to feed on host brood prior to the trial. This would afford the individual mites different nutritional starting points at the outset of the trial.

Brood cells were opened by removing the wax capping using dissecting tools. Foundress mites were transferred to artificial enclosures and given 20µl of honey bee tissue through an artificial membrane composed of a thin layer of parafilm. Survivorship was recorded once per day over the course of 7 days. At the start of the trial, each foundress mite was introduced into her own artificial enclosure, a standard commercial queen cup made of compressed wax. Inside of each cell, we introduced one size five gelatin capsule containing the treatment solution. Size five gelatin capsules are 1.1 cm in length, about the size of an average 5th instar worker bee larva and functioned well as “decoy larva” for this experiment. A large section of the gelatin capsule was cut away and replaced with Parafilm stretched by hand to between 10 and 15 µm. Bruce et al. (1988) (2) showed that mites were able to feed through a membrane of this thickness. Live pre-pupae were pressed against the parafilm membrane to transfer cuticular semiochemicals onto the surface of the membrane to stimulate feeding

in foundress mites. Parafilm was used to coat the outside of the cell to give a similar consistency to that of soft larva. As a wax product, parafilm adheres very well to the surface of other wax products, thus to hold the pill in place, we pressed the parafilm coating the outside of the pill against the roof of the wax enclosure. The inside of the cell was wrapped in parafilm as well to avoid the pill dissolving when exposed to the diet (SI Appendix, Fig. S4). The foundress mite (and any offspring) were allowed to feed ad libitum through this membrane for the duration of the trial. The contents of the capsule were filled once every 24 hours by piercing the wall of the capsule with a hypodermic needle and injecting the treatment solution into it directly. Artificial cells containing the foundress mite and the decoy larva were pressed into standard microcentrifuge tube racks and placed in an incubator at 34°C and 70% RH.

Five foundress mites were randomly assigned to each treatment per trial and 3 trials were conducted. Treatment solution consisted of 1 of the following formulations: 75% hemolymph to 25% fat body, 25% hemolymph to 75% fat body, 50% hemolymph to 50% fat body by volume, 100% hemolymph, or 100% fat body in DPBS (Dulbecco's phosphate buffered saline to function as a carrier mimicking the salinity and pH of insect hemolymph). Hemolymph was collected from nurse bees and transferred into pills using *Hamilton* Gastight Chromatography Syringes. Fat body tissue for this trial was dissected from the same nurse bees. While immature bees are the natural host of gravid *Varroa*, the distribution of fat body cells as a free-floating mass throughout the hemolymph made it prohibitively difficult to adequately separate the two tissues for a clear assay. Adult fat body was used because it is a connected mass localized to the inner dorsal surface, and to a greater extent, the inner ventral surface of the metasoma. After removing the digestive system, and wicking away excess fluid, fat body tissue was removed using fine forceps. This tissue was liquefied using a Polytron PT 1300D hand-held tissue homogenizer. and transferred into the pills using *Hamilton* Gastight Chromatography Syringes. Nurse bees were collected directly from the brood nest of several colonies tended by the University of Maryland, College Park. In addition, our diet relied of fat body from adult honey bees.

Microscopy

Low Temperature Scanning Electron Microscopy

Low temperature scanning electron microscopy was conducted at the USDA ARS Electron and Confocal Microscopy Unit using techniques outlined by Bolton et al. (2014) (3) and Ramsey et al. (2018) (4). *Varroa* parasitized honey bee metasoma were dissected to expose the feeding mite. Each metasoma was secured to 15 cm x 30 cm copper plates using ultra smooth, round (12mm diameter), carbon adhesive tabs (Electron Microscopy Sciences, Inc., Hatfield, PA, USA). The specimens were frozen conductively, in a Styrofoam box, by placing the plates on the surface of a pre-cooled (-196 °C) brass bar, the lower half of which was submerged in liquid nitrogen (LN2). After 20-30 seconds, the holders containing the frozen samples were transferred to a Quorum PP2000 cryo-prep chamber (Quorum Technologies, East Sussex, UK) attached to an S-4700 field emission scanning electron microscope (Hitachi High Technologies America, Inc., Dallas, TX, USA). The specimens were etched inside the cryo-transfer system to remove any surface contamination (condensed water vapor) by raising the temperature of the stage to -90 °C for 10-15 min. Following etching, the temperature inside the chamber was lowered below -130 °C, and the specimens were coated with a 10nm layer of platinum using a magnetron sputter head equipped with a platinum target. The specimens were transferred to a pre-cooled (-130 °C) cryostage in the SEM for observation. An accelerating voltage of 5kV was used to view the specimens. Images were captured using a 4pi Analysis System (Durham, NC).

Axiozoom Fluorescent Microscopy

A *Zeiss AxioZoom V16* stereo zoom microscope (Thornwood, NY) was used to obtain images. The images were observed using a 1x 0.25NA or 2.3X 0.25NA PlanNeoFluor objective. LED lighting was used for brightfield imaging and an AxioCam HRC Color camera was used to capture the images. Fluorescence microscopy was accomplished using a 200-watt mercury vapor lamp (HXP Short Arc Lamp, Thornwood,

NY) with a filter set for YFP with excitation at 500nm, beam splitter 515nm and emission at 535nm; mRFP with excitation at 572nm, beam splitter 590nm and emission at 600nm. Fluorescence was captured using an AxioCam 506 mono camera. Zen 2 Pro Blue (Thornwood, NY) 64-bit software was used to capture and preprocess images.

Confocal Laser Scanning Microscopy

A Zeiss LSM710 confocal laser scanning microscopy (CLSM) system was utilized as detailed by Ramsey et al. (2018) (4). The samples were mounted on slides and observed using a Zeiss Axio Observer™ (Thornwood, NY) inverted microscope with 10x 0.5 NA and 40x 1.2 NA Plan-Apochromat objectives. Several excitation wavelengths were utilized, 405nm (DAPI), 488nm (GFP), 515 (YFP) and 561nm (DsRed) with corresponding filter sets for each emission, 410-484nm (DAPI), 494-554nm (GFP), 530-555 (YFP) and 566-704nm (DsRed) with a pin hole of 33µm. Zeiss Zen 2012 Pro software was used to obtain 20-50 z-stack images to produce maximum intensity projections.

Transmission Electron Microscopy

Adult bees were taken from Varroa infested colonies, and a single drop of cyanoacrylate glue was placed on actively feeding mites. The glue permanently affixed the position of the mite between the tergite and/or sternite of the bee. The head and thorax of the bee was removed and the abdomen with attached mite was fixed in 2.5% glutaraldehyde, 0.05M sodium cacodylate for 2 hours. After initial fixation, the abdomen was further dissected and trimmed to a size encompassing the area directly around the mite, and a small cut was made in the posterior end of the mite to facilitate fixation and embedding. The bee/mite complex was fixed at 4°C overnight, then the tissue was rinsed 5 times with 0.05M cacodylate buffer and post-fixed in 1% buffered osmium tetroxide for 2 hours at room temperature. The tissue was then rinsed 5 times in the same buffer, dehydrated in a graded series of

ethanol followed by 2 exchanges of propylene oxide, infiltrated in a graded series of LX-112 resin/propylene oxide and polymerized in LX-112 resin at 65°C for 24 hrs. Silver-gold sections (60-90nm) were cut on a Reichert/AO Ultracut ultramicrotome with a Diatome diamond knife and mounted onto formvar-coated copper slot grids. Grids were stained with 4% uranyl acetate and 3% lead citrate and imaged at 80kV with a Hitachi HT-7700 transmission electron microscope. For light microscopy, 0.5µm thick sections were mounted onto glass slides, stained with toluidine blue, and imaged with a *Zeiss Axiozoom V16* stereo zoom microscope.

-Statistical Analysis

The significance of mite spatial preference was tested using a generalized linear model (glm). Comparison of mite levels on the left and right side of the honey bees was conducted via chi-square analysis in which equal frequencies on each side was the a priori expectation (mites on the thorax were not included in this analysis). The proportions representing the fluorescence profiles of biostained fat body, hemolymph, and Varroa were analyzed via Proportion Test (prop test). Survivorship curves were compared to progressively simpler Weibull distributions and afforded a statistical penalty for complexity. Models were tested and adjusted for non-constant hazard and the potential trial effect. Models were compared using a standard Analysis of Variance (ANOVA). Egg production was tested by deletion and analyzed via ANOVA as well. All statistical analyses were performed using R statistical computation software version 3.4.2 at default settings.

References

1. Garedew A, Schmolz E, & Lamprecht I (2004) The energy and nutritional demand of the parasitic life of the mite Varroa destructor. *Apidologie* 35(4):419-430.

2. Bruce W, Chiesa F, Marchetti S, & Griffiths D (1988) Laboratory feeding of Varroa jacobsoni Oudemans on natural and artificial diets (Acari: Varroidae). *Apidologie* 19(2):209-218.

3. Bolton SJ, Klompen H, Bauchan GR, & Ochoa R (2014) A new genus and species of Nematalycidae (Acari: Endeostigmata). *Journal of Natural History* 48(23-24):1359-1373.

4. Ramsey S, Gulbronson CJ, Mowery J, Ochoa R, & Bauchan G (2018) A Multi-Microscopy Approach to Discover the Feeding Site and Host Tissue Consumed by Varroa destructor on Host Honey Bees. *Microscopy and Microanalysis* 24(S1):1258-1259.
